# Association between the early mobilization of mechanically ventilated patients and independence in activities of daily living at hospital discharge

**DOI:** 10.1038/s41598-023-31459-1

**Published:** 2023-03-14

**Authors:** Shinichi Watanabe, Jun Hirasawa, Yuji Naito, Motoki Mizutani, Akihiro Uemura, Shogo Nishimura, Yasunari Morita, Yuki Iida

**Affiliations:** 1 Department of Rehabilitation Medicine, National Hospital Organization, Nagoya Medical Center, 4-1-1 Sannomaru, Naka-ku, Nagoya, Aichi 460-0001 Japan; 2 Department of Physical Therapy, Faculty of Rehabilitation, Gifu University of Health Science, Gifu, Gifu Japan; 3 Department of Rehabilitation Medicine, Tosei General Hospital, Seto, Aichi Japan; 4 Department of Rehabilitation Medicine, National Hospital Organization, Shizuoka Medical Center, Nagasawa, Shimizu, Suntougun, Shizuoka Japan; 5 Department of Rehabilitation Medicine, Ichinomiyanishi Hospital, Kaimeitaira, Itinomiya, Aichi Japan; 6 Department of Rehabilitation, Toyohashi Municipal Hospital, Hachikennishi, Aotake, Toyohashi, Aichi Japan; 7 Department of Rehabilitation Medicine, Kainan Hospital, Namihonden, Maegasu, Yatomi, Aichi Japan; 8 Department of Emergency Medicine, National Hospital Organization, Nagoya Medical Center, Sannomaru, Nakaku, Nagoya, Aichi Japan; 9 Department of Physical Therapy, School of Health Sciences, Toyohashi Sozo University, 20-1 Matushita, Ushikawa-cho, Toyohashi, Aichi 440-8511 Japan

**Keywords:** Disease prevention, Therapeutics, Geriatrics, Risk factors

## Abstract

Physical dysfunction after discharge from the intensive care unit (ICU) is recognized as a common complication among ICU patients. Early mobilization (EM), defined as the ability to sit on the edge of the bed within 5 days, may help improve physical dysfunction. However, the barriers to, and achievement of, EM and their impact on physical dysfunction have not been fully investigated. This study aimed to investigate the achievement of EM and barriers to it and their impact on patient outcomes in mechanically ventilated ICU patients. We conducted this multicenter retrospective cohort study by collecting data from six ICUs in Japan. Consecutive patients who were admitted to the ICU between April 2019 and March 2020, were aged ≥ 18 years, and received mechanical ventilation for > 48 h were eligible. The primary outcome was the rate of independent activities of daily living (ADL), defined as a score ≥ 70 on the Barthel index at hospital discharge. Daily changes in barriers of mobilization, including consciousness, respiratory, circulatory, medical staff factors, and device factors (catheter, drain, and dialysis), along with the clinical outcomes were investigated. The association among barriers, mobilization, and Barthel index ≥ 70 was analyzed using multivariable logistic regression analysis. During the study period, 206 patients were enrolled. EM was achieved in 116 patients (68%) on the fifth ICU day. The primary outcome revealed that achieving EM was associated with a Barthel index ≥ 70 at hospital discharge [adjusted odds ratio (AOR), 3.44; 95% confidence interval (CI), 1.70–6.96]. Device factors (AOR, 0.31; 95% CI, 0.13–0.75, respectively) were significantly associated with EM achievement. EM was associated with independent ADL at hospital discharge. Time to first mobilization and barriers to achieving mobilization can be important parameters for achieving ADL independence at discharge. Further research is required to determine the most common barriers so that they can be identified and removed.

## Introduction

Dramatic developments and improvements in the technology, equipment, and educational systems used in intensive care units (ICU) have reduced mortality among critically ill patients over the past four decades^1^. However, the proportion of patients with severe physical disorders has also increased concomitantly^2^. Physical disability occurs in 40–70% of ICU survivors^3–5^ and can last for several months or years after hospital discharge^6^. Critically ill patients admitted to the ICU have poor general conditions and tend to be immobilized, especially with mechanical ventilation management^7^. Independence in activities of daily living (ADL) is considered one of the most important factors for returning home after an ICU stay^8,9^. Active physical rehabilitation during ICU stays, especially when initiated within the first 72 h, is recommended to prevent physical disabilities and improve the clinical outcomes of ICU patients^10^.

Previous studies have shown that initiating early mobilization (EM) after ICU admission reduces the incidence of ICU-acquired weakness (ICU-AW) and delirium^4,11^, length of ICU and hospital stays^12,13^, duration of mechanical ventilation^4,14^, and medical costs^15,16^ while improving quality of life^17^. Other studies have revealed that achieving mobilization, such as sitting on the edge of the bed, standing, or walking early during the ICU stay, may improve outcomes^4,11–15,18^. For example, delays in mobilization for > 5 days after admission to the ICU can be detrimental^16^. Therefore, it is necessary to develop an efficient method for achieving EM in ICU patients. Notably, the term “mobilization” indicates the ability to sit on the edge of the bed, and “EM” indicates the time to achieve mobilization within 5 days of ICU admission. These definitions were based on previous studies^16,19^.

EM in critically ill patients is expected to have many effects, but there are several barriers to its actual implementation^20,21^. The main barriers shown in previous studies are deep sedation, a lack of coordination with rehabilitation-related professionals and other rehabilitation staff and team leaders, and a lack of understanding of the benefits and knowledge of early rehabilitation^20–23^. Therefore, even if the sedative management is of good quality, it is difficult to conduct effective rehabilitation^20–23^. However, improving the barriers and achieving EM can improve the physical function of patients and shorten the length of stay in the ICU and hospital^24^.

EM benefits have not been fully evaluated in terms of day-to-day changes in barriers to the implementation of EM, especially in multicenter studies^25^. A multicenter study is warranted to reduce any bias generated by particular features of any one research institute and the likely unique background of its patients. Investigating changes in the rate of mobilization and associated barriers simultaneously may guide planning rehabilitation, allowing these patients to achieve EM and prevent delays in initiating EM. In this study, we hypothesized that achieving EM in the ICU would improve patient outcomes. The primary objective of this multicenter study was to investigate the association between achieving EM in the ICU and ADL independence at hospital discharge. The secondary objective of this multicenter study was to identify barriers to the mobilization of patients and assess the association of barriers to EM on a day-to-day basis.

## Methods

### Study design and setting

The medical records of patients admitted to the ICU of one of six Japanese tertiary hospitals between April 2019 and March 2020 were retrospectively reviewed. All these were mixed medical-surgical ICUs. This multicenter retrospective cohort study was conducted at the Nagoya Medical Center and five other participating hospitals (Tosei General Hospital, Kainan Hospital, Itinomiyanishi Hospital, Toyohashi Municipal Hospital, and Shizuoka Medical Center). Detailed characteristics of the institutions are listed in Supplementary Table 1.

We followed the Strengthening the Reporting of Observational Studies in Epidemiology (STROBE) guidelines^26^ and all methods in this study were performed following the relevant guidelines and regulations. ICU patients who received mechanical ventilation for ≥ 48 h were screened for inclusion. The exclusion criteria were as follows: patients aged < 18 years, unable to walk independently before hospitalization^3^, neurologically impaired, incapable of communicating in Japanese, with a condition that limits mobilization, with a terminal/end-of-life status, or who died during ICU stay (Supplementary Table 2).

### Early mobilization protocol

This study’s early goal-directed rehabilitation protocol^3,10,13,15,16,25^ was developed > 6 months prior to study initiation, and the details of the protocol were specifically arranged according to the participating hospital. This protocol has been used in routine practice in multiple centers, and validation of the protocol’s safety has already been reported^27^. In this study, we sought to mobilize all patients equally and daily under the protocol tailored to each participating hospital. Details of the EM protocol are presented in Supplementary Table 3. The protocol includes five rehabilitation levels (level 1, passive range of motion and respiratory physical therapy; level 2, active range of motion; level 3, sitting exercise; level 4, standing exercise; and level 5, walking exercise) based on the patient’s medical condition. At each participating site, ICU physicians or physiotherapists referred to the protocol and decided each patient’s rehabilitation level based on the patient’s condition. All patients received at least one rehabilitation session per weekday for 20 min by a physical therapist. In addition, for weekend rehabilitation, at least one 20-min rehabilitation session was performed by the attending nurse using a protocol similar to that for the weekdays. The target number of implementation units and frequency were determined by each hospital based on the patient's condition.

All participating hospitals followed the 2018 Clinical Practice Guidelines^28^ and the clinical practice guidelines for the management of acute respiratory distress syndrome^29^. The former concerns the management of pain, agitation, and delirium in adult patients in the ICU, and the latter concerns ventilator settings and drug therapy. There was no difference in weekend medical treatment among the participating hospitals. After ICU discharge, physical or occupational therapists provided rehabilitation, such as muscle strengthening, balance, walking, and stair exercises, for more than 20 min on weekdays to each patient according to the rehabilitation policy in the general ward of each hospital.

### Data collection

Patient data were retrieved retrospectively from electronic medical records. Data on baseline characteristics of all enrolled patients were collected at the time of ICU admission, including age, sex, body mass index, Charlson comorbidity index, Barthel index before hospitalization, admission source, ICU admission diagnosis, Acute Physiology and Chronic Health Evaluation II (APACHE II) score, Sequential Organ Failure Assessment (SOFA) score, need for mechanical ventilation, continuous vasopressor use, continuous sedation, continuous analgesia, and hemodialysis. Barthel index before hospitalization was scored at the time of ICU admission based on the information obtained from the family or the patients if they were conscious. Vasopressors, sedation, and analgesia refer to those administered continuously beyond ICU admission and did not include intermittent administration.

Rehabilitation session data were recorded daily by a physical therapist or nurse following each rehabilitation session, including data on the highest activity level according to the ICU mobility scale^30^ and barriers preventing mobilization during that session. The ICU mobility scale is a sensitive 11-point ordinal scale, with scores ranging from 0 (no mobilization) to 10 (independent ambulation). We collected these data within the first 5 days of the ICU stay.

**Figure 1:**
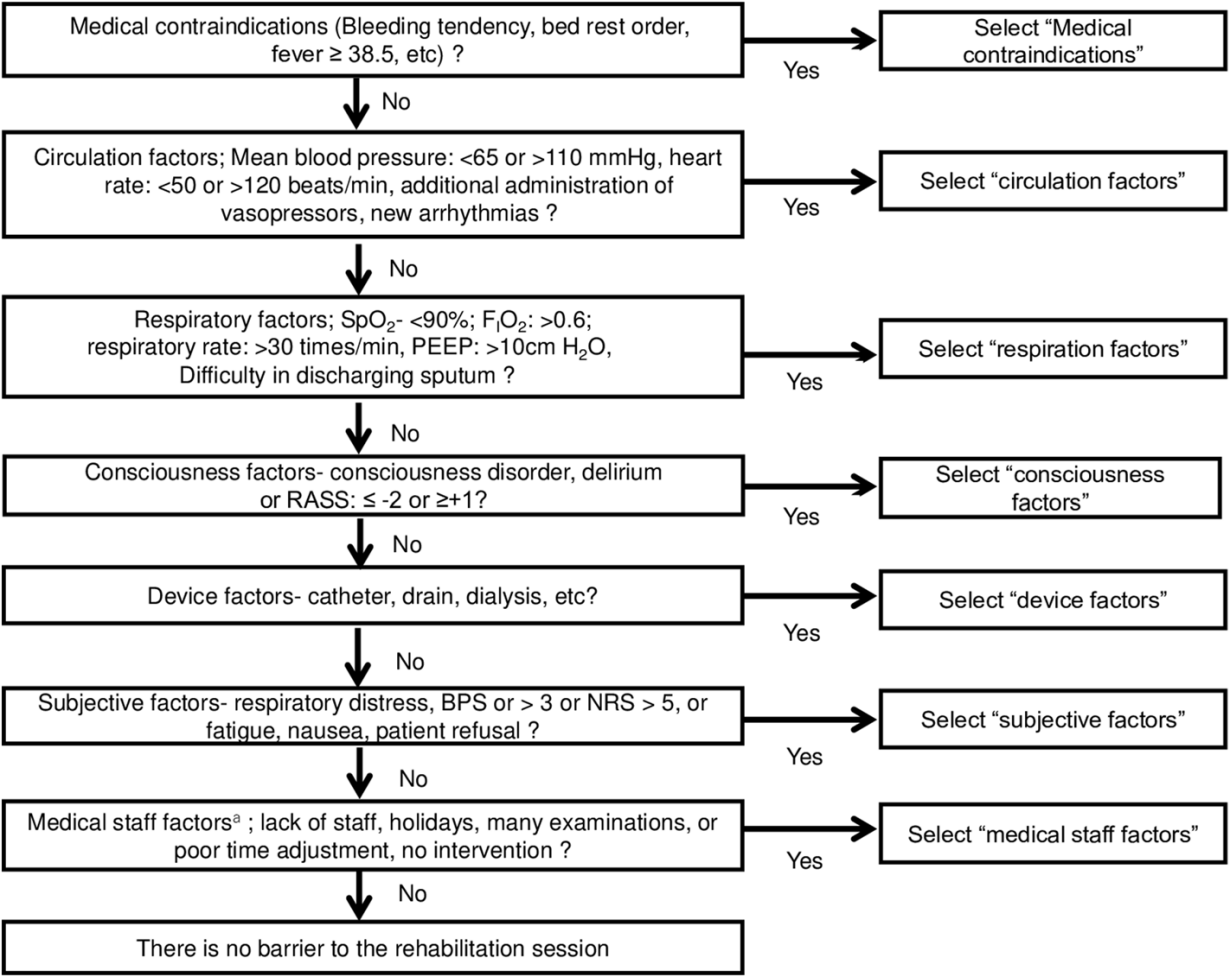
Flow chart of the patient selection process. *ICU* intensive care unit. Superscript a: neurological complications include cerebral infarction, cerebral hemorrhage, acute subdural hematoma, acute epidural hematoma, traumatic subarachnoid hemorrhage, and encephalitis. Superscript b: mental and cognitive diseases include depression, anxiety, schizophrenia, dementia, cerebral infarction, cerebral hemorrhage, dementia, and alcoholism. This includes cases where standard rehabilitation and assessment of outcomes are difficult to assess due to the inability to communicate in Japanese.

Perceived barriers included predefined barriers described in prior studies^21,22,24,25,31^. Barrier assessment was routinely used at each institution prior to this study’s initiation. These included consciousness; subjective symptoms; and respiratory, circulatory, device, subject, and medical staff factors^25^. Details of these factors are shown in Fig. 1. If several barriers were identified in one session, only the primary reason was recorded, and not the individual components of categories. During each rehabilitation session, a physical therapist or nurse in the ICU determined the primary barrier to preventing mobilization by the end of the session according to the algorithm shown in Fig. 1. The priority of this list was based on previous studies investigating barriers to mobilization, the consensus of early rehabilitation experts created in Japan, and our clinical experience^24,25,32,33^.

### Study outcomes

The primary outcome measured in this study was ADL independence at hospital discharge, which was defined as a score of 70 or higher on the Barthel index^4,7^. This widely used and reliable scale measures a patient's ability to perform daily activities^34^.

The secondary outcomes included total medical costs, duration of mechanical ventilation, duration of ICU stay, duration of hospital stay, the incidence of ICU-AW at ICU discharge, incidence of delirium during ICU stay, and rate of discharge to home. Data on medical costs and discharge destinations were collected from the Medical Affairs Department.

Medical costs were calculated based on the diagnostic procedure combination/per-diem payment system and converted from Japanese Yen to United States dollars (USD) at an exchange rate of 108 Yen/USD^35^. Delirium was assessed using The Intensive Care Delirium Screening Checklist^36^. ICU-AW was defined as a Medical Research Council sum score (evaluated by a physical therapist) < 48 at ICU discharge^37^.

### Statistical analysis

Continuous variables are presented as medians and interquartile ranges (IQRs), whereas categorical variables are presented as numbers and percentages. When appropriate, all patient outcomes were categorized as dichotomous data using median values^16^.

We performed multiple logistic regression analysis to evaluate the effect of EM on patient outcomes. The analysis took into account several factors such as age, Barthel index before hospitalization, planned operation, septic shock, APACHE II score, and use of continuous vasopressors. These variables were selected based on the results of previous studies^2,4,11,24,28,38,39^. We limited the number of covariates to six to prevent overfitting the model^40^. For the post hoc analysis, multiple logistic regression analysis was performed to examine the association between barriers and the achievement of EM; the results are reported as odds ratios (ORs) with 95% confidence intervals (CIs). In multiple logistic analysis, the explanatory variables included the following seven factors associated with mobilization barriers: medical contraindication and respiratory, circulatory, consciousness, device, subject, and medical staff factors from days 1 to 5. In our study, we used multivariable logistic regression analysis and Bonferroni correction to examine the relationship between mobilization barriers, primary and secondary outcomes, and achieving EM as measured by ADL independence at hospital discharge.

Three sensitivity analyses were performed. (1) The association between the achievement of mobilization and outcomes within 2, 3, 4, 6, or 7 days after ICU admission was examined using the same method (multiple logistic regression analysis) as the primary analysis. (2) We included those who died after ICU discharge in the ADL non-independent group because the association between EM and fatality is assumed to be bidirectional, and ADL non-independence can result from fatality. ADL non-independence may be associated with in-hospital mortality (3) To address other potential confounders of ADL independence, we selected different covariates to assess the robustness of our findings. In Model 2, sex, body mass index, and the Charlson comorbidity index were added. Model 3 incorporated institutions and SOFA scores. Variables in the model with P < 0.05 from the lack-of-fit test were excluded from the results of this study considering non-fitting^41^.

All analyses were performed using JMP software (version 13.0; SAS Institute Inc., Cary, NC, USA). Statistical tests were two-sided, and statistical significance was defined as P < 0.05.

### Ethics approval and consent to participate

This multicenter retrospective cohort study was approved by the Ethics Committee of Nagoya Medical Center (approval number: 2021-012) and the respective ethics committees of the five other participating hospitals. It was conducted in accordance with Helsinki Declaration and the need for informed consent, according to national legislation, was waived by the IRB listed above because this was a retrospective cohort study. Human participants' names and other identifiers were not used during the study process and were not included in all sections of the manuscript, including Supplementary Information.

## Results

### Baseline patient characteristics

**Figure 2:**
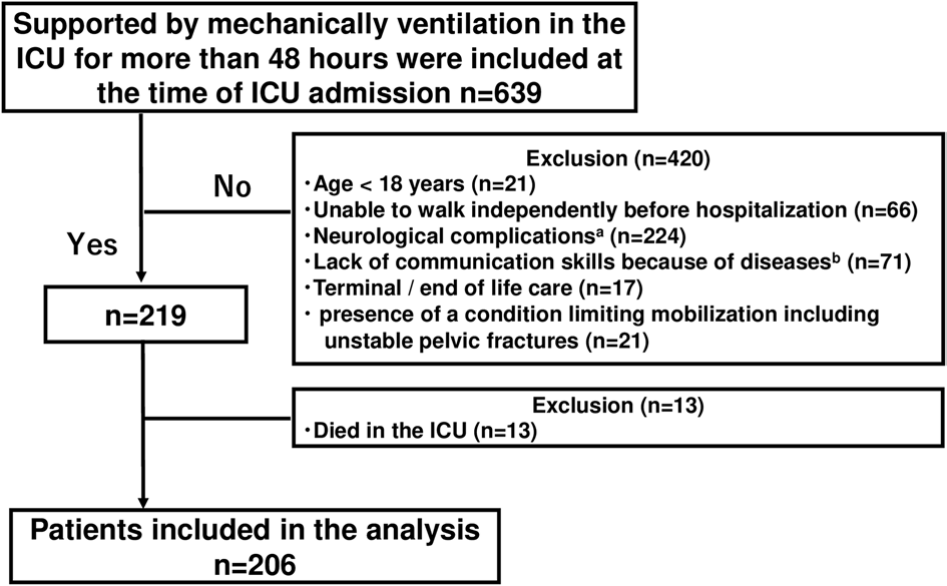
Algorithm to determine the primary barrier preventing mobilization. *SpO*_*2*_ oxygen saturation of the peripheral artery, *FiO*_*2*_ fraction of inspiratory oxygen, *RASS* Richmond agitation sedation scale, *BPS* behavioral pain scale, *NRS* numerical rating scale. The barrier to mobilization was determined by the intensivist in charge of the patient following this algorithm. In every rehabilitation session, only one selected barrier was recorded in the medical record.

During the study period from April 2019 to March 2020, 639 consecutive patients were screened, and 13 died during hospitalization. Finally, 206 patients without deficiencies were enrolled in this study (Fig. 2). The baseline characteristics of the enrolled patients are shown in Table 1. There were 139 men (68%), with a median age of 70 (IQR, 62–77) years. The median APACHE II and SOFA scores at ICU admission were 23 (IQR, 17–28) and 8 (IQR, 6–10), respectively (Table 1). A total of 115 patients (56%) achieved mobilization on or before the fifth day of ICU stay. The rates of achieving each mobilization level are shown in Supplementary Fig. 1. In this study, all 42 patients who were discharged from the ICU within fifth days achieved mobilization during their ICU stay.

**Table 1:** Baseline characteristics at the time of intensive care unit admissions.

Variables	All Patients, n = 206
Age (years), median [IQR]	70 [62–77]
Gender (male), n (%)	139 (68)
BMI (kg/m^2^), median [IQR]	22.7 [19.7–26.6]
Charlson Comorbidity Index, median [IQR]	1 [0–3]
Barthel index before hospitalization, n (%)^a^	100 [100–100]
Admission source, n (%)	
Emergency department	126 (61)
General ward in hospital	36 (18)
Planned operation^b^	44 (21)
Sepsis shock at ICU admission, n (%)	120 (58)
ICU admission diagnosis, n (%)	
Acute respiratory failure (including pneumonia)	34 (16)
Cardiovascular disease	87 (42)
Gastric or colonic surgery	44 (21)
Sepsis, non-pulmonary	29 (14)
Other diagnoses	12 (6)
APACHE II score, median [IQR]	23 [17–28]
SOFA at ICU admission, median [IQR]	8 [6–10]
The use of continuous vasopressor during ICU stay, n (%)	148 (72)
The use of continuous sedation during ICU stay, n (%)	201 (98)
The use of continuous analgesia during ICU stay, n (%)	183 (89)
The use of dialysis during ICU stay, n (%)	43 (21)
The use of neuromuscular blocking agent during ICU stay, n (%)	35 (17)

Data are presented as median [interquartile range] or number (%).

*IQR* interquartile range, *BMI* body mass index; *ICU* intensive care unit, *APACHE* acute physiology and chronic health evaluation, *SOFA* sequential organ failure assessment.

^a^Barthel index before hospitalization was scored at the time of ICU admission based on the information from the family or the patients if they were conscious.

^b^Breakdown of post-operation: cardiovascular surgery, 27 (61%); Gastrointestinal surgery, 11 (25%); other surgery, 6 (14%).

### Primary and secondary outcomes

The prevalence of ADL non-independence at hospital discharge was 30.1% (62/206) (Table 2). Table 2 shows the association between mobilization and outcomes. After adjusting for covariates, the primary outcome revealed that achieving EM was associated with ADL independence at hospital discharge [adjusted odds ratio (AOR), 3.44; 95% CI, 1.70–6.96]. Results regarding the secondary outcomes also showed a significant association with EM achievement and total medical costs (AOR, 3.05; 95% CI, 1.59–5.87), duration of ICU stay (AOR, 4.62; 95% CI, 2.47–8.66), and the duration of hospital stay (AOR, 2.98; 95% CI, 1.53–5.80). In the sensitivity analysis, our results were unaffected by altering the definition of EM (Supplementary Table 4), the inclusion of those who died after ICU discharge (Supplementary Table 5), or by developing other models using different covariates (Supplementary Table 6). In the multivariable logistic regression analysis when varying the definitions of EM within 2, 3, 4, or 6 days, the highest odds ratio between achieving EM within 5 days and ADL independence at hospital discharge was recorded (Supplementary Table 4).

**Table 2:** Multivariable logistic regression analysis of the association between early mobilization achievement and outcomes.

Outcomes	All (n = 206)	Adjusted OR (95% CI)	p-value
Primary outcomes			
ADL independence at discharge, n (%)	125 (61)	3.44 (1.70–6.96)	< 0.001
Secondary outcomes			
Total medical costs < 2500-dollar, n (%)	95 (49)	3.05 (1.59–5.87)	< 0.001
Duration of mechanical ventilation < 5 days, n (%)	106 (52)	1.38 (0.75–2.53)	0.295
ICU length of stay < 7 days, n (%)	105 (51)	4.62 (2.47–8.66)	< 0.001
Hospital length of stay < 28 days, n (%)	79 (38)	2.98 (1.53–5.80)	0.001
ICU-AW at ICU discharge, n (%)^a^	73 (45)	0.39 (0.19–0.81)	0.012
Delirium during ICU stay, n (%)^b^	78 (38)	0.44 (0.22–0.88)	0.023
Discharge to home, n (%)	144 (70)	2.26 (1.14–4.46)	0.019

Variables for the outcomes in the multivariable logistic regression included age, Barthel index before hospitalization, planned operation, Sepsis shock at ICU admission, APACHE II score, use of continuous vasopressor.

*OR* odds ratio*, IQR* interquartile range, *ADL* activity daily living, *ICU* intensive care unit, *APACHE* acute physiology and chronic health evaluation, *ICU-AW* ICU-acquired weakness.

^a^Of 206 patients, 44 were missing.

^b^Of 206 patients, 39 were missing.

### Barriers to mobilization

Medical contraindications were most frequently described as barriers to EM on days 3 (20%), 4 (16%), and 5 (18%). On days 1 and 2, the most frequently described barrier was circulatory factors (35% and 25%, respectively) (Table 3).

**Table 3:** Primary barriers preventing the achievement of early mobilization.

	Day 1 (n = 206)	Day 2 (n = 206)	Day 3 (n = 206)	Day 4 (n = 191)	Day 5 (n = 164)
Medical contraindication	67 (33)	45 (22)	**41 (20)**	**31 (16)**	**29 (18)**
Circulatory factor	**72 (35)**	**51 (25)**	28 (13)	15 (8)	9 (5)
Respiratory factor	17 (8)	23 (11)	23 (12)	15 (8)	13 (8)
Consciousness factor	24 (12)	32 (16)	27 (13)	27 (15)	19 (12)
Device factor	11 (5)	13 (6)	9 (4)	15 (8)	7 (4)
Subject factor	6 (3)	6 (3)	7 (3)	4 (2)	6 (3)
Medical staff factor	6 (3)	11 (5)	16 (8)	11 (5)	11 (7)
Achievement of EM	3 (1)	25 (12)	55 (27)	73 (38)	70 (43)

Number of patients (%).

*EM* early mobilization, *ICU* intensive care unit.

Significant values are in bold.

Among the seven major barriers detected within 5 days of ICU admission, we found that from days 1 to 5, device factors (AOR, 0.31; CI, 0.13–0.75) were significantly associated with EM achievement (Table 4). Supplementary Table 7 shows the number of components by category for each of the seven major barriers detected within 5 days of ICU admission.

**Table 4:** Association between mobilization barriers and achieving early mobilization.

Variable	Achieved EM		
	OR	95% CI	P value
Medical contraindication from day 1 to 5	0.54	0.28–1.05	0.070
Circulatory factor from day 1 to 5	0.56	0.30–1.07	0.081
Respiratory factor from day 1 to 5	0.44	0.21–0.92	0.029
Consciousness factor from day 1 to 5	0.59	0.32–1.12	0.108
Device factor from day 1 to 5	**0.31**	**0.13–0.75**	** < 0.001**
Subject factor from day 1 to 5	1.52	0.59–3.96	0.385
Medical staff factor from day 1 to 5	0.69	0.34–1.40	0.298

We adjusted for multiple comparisons by using the Bonferroni correction and considered a result to be statistically significant if P < 0.007.

*OR* odds ratio, *CI* confidence interval, *EM* early mobilization, *ADL* activity daily living, *ICU* intensive care unit.

Significant values are in bold.

## Discussion

In this multicenter retrospective cohort study conducted using data from patients treated in the ICUs of one of six hospitals in Japan, we investigated the achievement of EM in a mixed population of mechanically ventilated patients in the ICU as well as the association between EM and ADL independence at hospital discharge.

Moreover, this study reviewed the barriers preventing EM and ADL independence at multiple centers. This is the first multicenter study to investigate the relationship between day-to-day changes in barriers to EM and ADL independence at hospital discharge. Our findings suggest that within 5 days of ICU admission, removing barriers to mobilization for patients on mechanical ventilation and initiating mobilization are preferable in terms of preventing physical dysfunction in the ICU.

The underlying pathophysiological mechanisms of physical dysfunction at discharge are multifactorial, including age, altered level of consciousness, delirium, ICU-AW development, and immobility^3,4,24^. Of these, bed rest in the supine position is a risk factor that can be easily improved. Given these proposed mechanisms, EM against mechanical ventilation is a potentially efficient strategy, similar to how muscle strength exercise appears to prevent the development of ICU-AW^28,37^. However, some recent randomized studies have failed to detect significant improvements in the EM^42^, which may have been related to delayed EM initiation, beginning approximately after more than 1 week after ICU admission. The achievement of EM in this study was associated not only with ADL independence at hospital discharge but also with total medical costs, ICU stay duration, hospital stay duration, and the incidence of ICU-AW and delirium. Previous reports have also indicated that achieving EM within 5 days of ICU admission does not affect survival but is effective in improving functional outcomes^16,19^. Thus, achieving EM in the ICU, as shown in this study, might help prevent disuse syndrome and achieve independent ADL.

This study also showed daily changes in the rate of achieving mobilization, which was very low on ICU days 1 (1%) and 2 (12%) and increased from days 3 (27%) to 5 (43%). The overall rate of achieving mobilization (56%) was comparable with that in a prior study^22^. Medical restrictions and cardiovascular and respiratory factors were identified in more than half of the patients as the main barriers to achieving mobilization on days 1 and 2 in the ICU. Most patients were probably hemodynamically unstable upon admission to the ICU and required vasopressor support. However, in this study, medical and circulatory factors were not significantly associated with ADL independence at hospital discharge. Future studies should consider whether low and passive exercise^43^ or neuromuscular electrical stimulation^44^ can substitute for EM in patients who cannot achieve EM due to medical contraindications or cardiovascular factors. Previous studies have reported that factors related to the medical staff are a major barrier to mobilization^21–23^. However, in this study, its influence was very small, and as a result, the achievement rate of EM was high. Based on established protocols, assessing barriers to EM may reduce medical staff-related factors. The results of this study were drawn from data from a hospital that actively performs ICU rehabilitation in Japan, and may not be generalizable to all ICUs.

When examining the relationship between ADL independence at hospital discharge and the time to the first mobilization varied by 2, 3, 4, 6, or 7 days after ICU admission, the value at 5 days showed the highest odds ratio. Shortening the interval to achieve EM after overcoming barriers may be an important aspect of early rehabilitation to maximize the impact on mechanical ventilation outcomes. The significant association between EM achievement and the Barthel index at discharge under multiple conditions supports our theory.

The small sample size and comparability of the two groups are central limitations. These could limit the generalizability to other ICUs. There are unadjusted confounding factors such as nutrition and ventilator settings. However, to do our best, multivariable analysis using logistic regression analysis tuned by key clinical and potential confounding factors showed consistent results. However, in the logistic regression analysis adjusted for potential confounders, covariates may have exhibited overfitting, and these results should be interpreted with caution. In our study, the outcomes were limited within short-term outcomes. Additionally, in this study, whether the patient could receive rehabilitation at the level of sitting over the edge or higher were depending on the rehabilitation policy in each participating hospital. Furthermore, in this study, we were unable to investigate the number of implementation units and frequency of rehabilitation implemented at each hospital. Therefore, it was difficult to identify whether EM could not be provided due to poor general conditions or other factors. The algorithm for determining barriers to mobilization in this study was created based on our clinical experience, and its reliability and validity were not sufficiently verified. A multicenter, prospective, cohort study including all mechanically ventilated patients will likely validate the questions that remain unanswered.

The main barriers to mobilization were device factors. Our observations show that the initiation of physical rehabilitation with an intensity greater than that needed for sitting on the edge of the bed within 5 days of ICU admission appears to be the preferred strategy for improving ADL independence at hospital discharge. Overcoming the barrier of mobilization may also be necessary to improve the ADL independence rate at hospital discharge. Further research is required to validate our results.

## Supplementary Information

Supplementary Information.

## Data Availability

The data will be available with the corresponding author at demand.
