# Supplementary material for: Association between the early mobilization of mechanically ventilated patients and independence in activities of daily living at hospital discharge
Source: Sci Rep. 2023 Mar 14;13:4265. doi: 10.1038/s41598-023-31459-1 (PMC10015081; doi:10.1038/s41598-023-31459-1)
Supplement: Supplementary file 1 — Supplementary Information. [file 41598_2023_31459_MOESM1_ESM.docx]

**Supplementary Table 1.** **Characteristics of each institution and intensive care unit**

| Institutions | 1: Nagoya Medical Center | 2: Tosei General Hospital | 3: Shizuoka Medical Center | 4: Itinomiyanishi Hospital | 5: Toyohashi Municipal Hospital | 6: Kainan Hospital | Mean (SD) |
| --- | --- | --- | --- | --- | --- | --- | --- |
| No. of hospital beds, n | 740 | 633 | 450 | 465 | 800 | 540 | 604.7 (144.8) |
| No. of ICU beds, n | 6 | 8 | 10 | 8 | 6 | 8 | 7.7 (1.5) |
| No. of intensivists, n | 3 | 2 | 2 | 8 | 2 | 8 | 4.2 (2.9) |
| No. of PT in the hospital, n | 26 | 23 | 16 | 86 | 25 | 23 | 33.2 (26.1) |
| No. of dedicated PT in the ICU, n | 1 | 0 | 0 | 2 | 0 | 0 | 0.5 (0.8) |
| No. of nurses in the ICU, n | 24 | 33 | 35 | 24 | 23 | 25 | 27.3 (5.2) |
| Patient to nurse ratio | 2:1 | 2:1 | 2:1 | 2:1 | 2:1 | 2:1 | ― |
| System of ICU, yes or no | | | | | | |  |
| Mixed medical-surgical ICU | Yes | Yes | Yes | Yes | Yes | Yes | ― |
| Closed ICU: intensivists have the primary responsibility of treatment to the patients | No | No | No | Yes | No | No | ― |
| Mandatory consultant to intensivists at all ICU admission | Yes | Yes | No | No | No | Yes | ― |
| ICU physician is available for consultation about the patients | No | Yes | Yes | No | Yes | No | ― |

*SD =* standard deviation; *ICU* = Intensive Care Unit; *PT* = Physical Therapist.

**Supplementary Table 2. Exclusion criteria**

| **Exclusion Criteria** | **Details** |
| --- | --- |
| Age <18 years |  |
| Unable to walk independently before ICU admission | Patients who require a wheelchair other than a cane or other walking assistance prior to admission or help from others to walk were considered unable to walk independently. |
| Neurological complications | Neurological complications include cerebral infarction, cerebral hemorrhage, acute subdual hematoma, acute epidural hematoma, traumatic subarachnoid hemorrhage, and encephalitis. |
| Lack of communication skill in Japanese | Mental and cognitive diseases include depression, anxiety, schizophrenia, dementia, cerebral infarction, cerebral hemorrhage, dementia, and alcoholism. |
| Presence of a condition limiting mobilization including unstable pelvic fractures | Patients who were not allowed to out of bed mobilization for more than a week after admission the ICU due to fractures or trauma. |
| In terminal state or end of life |  |

*ICU* = Intensive Care Unit.

**Supplementary Table 3. Early Mobilization Protocol**

| **Level 1 Respiratory**  RASS−5 ～ －3 | **Level 2 HOB**  RASS ≥ −3 | **Level 3 Sitting**  RASS ≥ −1 | **Level 4 Standing**  RASS ≥ 0 | **Level 5 Walking**  RASS ≥ 0 |
| --- | --- | --- | --- | --- |
| **Physical therapy**  Passive ROM exercise  Respiratory physical therapy | **Physical therapy**  Positioning  Passive ROM exercise  Active ROM exercise  Respiratory physical therapy  continuous lateral rotation therapy | **Physical therapy**  Positioning  Passive ROM exercise  Active ROM exercise  Sitting on the edge of bed  Rising from the supine position | **Physical therapy**  Positioning  Passive ROM exercise  Active ROM exercise  Standing at side of bed  Stand and pivot to a chair | **Physical therapy**  Positioning  Passive ROM exercise  Active ROM exercise  Walk with assistance  Walk independently |
| **Positioning**  Posture change  HOB ≤45 degrees | **Positioning**  Posture change  HOB ≥60 | **Positioning**  Posture change  HOB ≥60 | **Positioning**  Posture change  HOB ≥60 | **Positioning**  Posture change  HOB ≥60 |
| **Step up criterion**  Oxygenation/ hemodynamic stability  Can withstand posture change  Can withstand HOB ≤45 degrees | **Step up criterion**  Can withstand supplementary motion of physical therapy  Can withstand HOB ≤60 degrees  Anti-gravity movement possible | **Step up criterion**  Can endure the active movement of physical therapy  Can withstand HOB ≤60 degrees  Can withstand sitting on the edge bed | **Step up criterion**  All exercise can be carried out  Can withstand partial weight standing | **Step up criterion**  Increase walking distance gradually |
| **Step up criterion to level 3 or higher are defined as**  RASS: -2 to +1, BPS ≤ 3 or NRS ≤ 5, SpO_2_ ≥ 90%, FIO_2_ < 0.6, PEEP < 10 cmH_2_O, respiratory rate: <35 times / min, mean blood pressure ≥ 65 mmHg, heart rate: 50 to 120 times / min, there were no new arrhythmias, no additional administration of vasopressors, no bleeding, no wound with the possibility of separation, no unstable fracture. | | | | |

*RASS,* Richmond agitation sedation scale; *ROM,* range of motion; *HOB*, head of bed; *BPS*, behavioral pain scale; *NRS*, numeric rating scale; *FIO_2_*, fraction of inspiratory oxygen; *PEEP,* positive end-expiratory pressure; EM, early mobilization.

**Supplementary Table 4. Relationship between achieving early mobilization per different definitions and activities of daily living independence at discharge**

| Variable | The definition of ICU Day in early mobilization | | | | | |
| --- | --- | --- | --- | --- | --- | --- |
|  | Within 2 days | Within 3 days | Within 4 days | Within 5 days | Within 6 days | Within 7 days |
| Adjusted model  ADL independence at discharge | **2.72**  **0.80**–**9.20** | **2.41**  **1.15**–**5.06** | **2.31**  **1.18**–**4.54** | **3.44**  **1.70-6.96** | **2.58**  **1.21–4.76** | 1.46  0.62–3.41 |

Data in table are presented as Odds Ratio (95% Confidence interval)

ICU= Intensive Care Unit, ADL= Activity daily living

Adjusted model: Multiple logistic regression analysis was performed to determine the primary outcome with the following covariates: age, sex, acute physiology and chronic health evaluation score, sequential organ failure assessment at the time of extubation, and use of continuous vasopressors, which were considered as factors related to the primary outcome.

| Outcomes | All (n=186) | Adjusted OR (95% CI) | p-value |
| --- | --- | --- | --- |
| Primary outcomes | | | |
| ADL independence at discharge, n (%) | 125 (66) | 3.99 (1.86–8.61) | <0.001 |
| Secondary outcomes | | | |
| Total medical costs < 2500-dollar, n (%) | 87 (50) | 2.61 (1.31–5.20) | 0.006 |
| Duration of mechanical ventilation < 7 days, n (%) | 139 (74) | 7.30 (3.21–16.63) | <0.001 |
| ICU length of stay < 7 days, n (%) | 102 (54) | 5.69 (2.89–11.22) | <0.001 |
| Hospital length of stay < 28 days, n (%) | 70 (37) | 2.36 (1.18–4.72) | 0.016 |
| ICU-AW at ICU discharge, n (%) ^a^ | 63 (42) | 0.45 (0.21–0.96) | 0.039 |
| Delirium during ICU stay, n (%) ^b^ | 69 (46) | 0.35 (0.17–0.74) | 0.006 |
| Discharge to home, n (%) | 144 (76) | 2.76 (1.27–5.98) | 0.010 |

**Supplementary Table 5. Multivariable logistic regression analysis of the association between early mobilization achievement and outcomes, excluding fatal cases**

*OR,* odds ratio*; IQR*, interquartile range; *ADL*, activities of daily living; *ICU*, intensive care unit; *APACHE*, Acute Physiology and Chronic Health Evaluation; *ICU-AW*, ICU-acquired weakness. Variables for the outcomes in the multivariable logistic regression included age, Barthel index before hospitalization, planned operation, septic shock at ICU admission, APACHE II score, and use of continuous vasopressors.

a. Of 186 patients, 42 were missing.

b. Of 186 patients, 37 were missing.

**Supplementary Table 6. Multivariable logistic regression analysis of the association between early mobilization achievement and outcomes, adjusted for different covariates**

| Outcomes | Model 2  Adjusted OR (95% CI) | p-value | Model 3  Adjusted OR (95% CI) | p-value |
| --- | --- | --- | --- | --- |
| Primary outcomes | | | | |
| ADL independence at discharge, n (%) | 3.19 (1.54–6.57) | 0.002 | 3.35 (1.51–7.39) | 0.003 |
| Secondary outcomes | | | | |
| Total medical costs < 2500-dollar, n (%) | 3.14 (1.61–6.13) | <0.001 | 1.96 (0.76–5.02) | 0.162 |
| Duration of mechanical ventilation < 7 days, n (%) | 6.37 (2.99–13.57) | <0.001 | 6.30 (2.84–13.95) | <0.001 |
| ICU length of stay < 7 days, n (%) | 4.85 (2.54–9.24) | <0.001 | 6.80 (3.27–14.16) | <0.001 |
| Hospital length of stay < 28 days, n (%) | 2.87 (1.47–5.60) | 0.002 | 3.84 (1.81–8.15) | <0.001 |
| ICU-AW at ICU discharge, n (%) ^a^ | 0.40 (0.19–0.84) | 0.016 | 0.42 (0.20–0.91) | 0.028 |
| Delirium during ICU stay, n (%) ^b^ | 0.46 (0.23–0.92) | 0.027 | 0.36 (0.16–0.78) | 0.010 |
| Discharge to home, n (%) | 2.12 (1.06–4.23) | 0.033 | 2.97 (1.37–6.41) | 0.006 |
| In-hospital mortality, n (%) | 1.19 (0.39–3.21) | 0.835 | 0.79 (0.24–2.59) | 0.701 |

*OR,* odds ratio*; IQR*, interquartile range; *ADL*, activities of daily living; *ICU*, intensive care unit; *APACHE*, Acute Physiology and Chronic Health Evaluation; *ICU-AW*, ICU-acquired weakness; *BMI,* body mass index; SOFA, Sequential Organ Failure Assessment. Model 2: Variables for the outcomes in the multivariable logistic regression included age, Barthel Index before hospitalization, planned operation, septic shock at ICU admission, APACHE II score, use of continuous vasopressors, sex, BMI, and Charlson Comorbidity Index. Model 3: Variables for the outcomes in the multivariable logistic regression included age, Barthel Index before hospitalization, planned operation, septic shock at ICU admission, APACHE II score, use of continuous vasopressors, sex, BMI, Charlson Comorbidity Index, institutions, and SOFA score.

a. Of 206 patients, 44 were missing.

b. Of 206 patients, 39 were missing.

**Supplementary Table 7. Number of components per category for each of the seven major barriers to mobilization detected within 5 days of intensive care unit admission**

| Variable | Number of times (%) |
| --- | --- |
| Medical contraindication from day 1 to 5 | **131** |
| Bleeding tendency | 21 (16) |
| Bed rest order | 93 (71) |
| Fever > 38.5 | 15 (11) |
| Circulatory factor from day 1 to 5 | **263** |
| Additional administration of vasopressors | 86 (33) |
| Mean blood pressure: <65 or >110 mmHg | 164 (62) |
| Heart rate: <50 or >120 beats/min | 7 (3) |
| New arrhythmias | 6 (2) |
| Respiratory factor from day 1 to 5 | **113** |
| F_I_O_2_: >0.6 | 33 (29) |
| SpO2- <90% | 31 (27) |
| Respiratory rate: >30 times/min | 11 (10) |
| PEEP: >10 cmH_2_O | 9 (8) |
| Difficulty in discharging sputum | 29 (26) |
| Consciousness factor from day 1 to 5 | **161** |
| Consciousness disorder | 27 (17) |
| Deep sedative | 110 (68) |
| Delirium | 24 (15) |
| Device factor from day 1 to 5 | **60** |
| Catheter | 33 (55) |
| Dialysis | 27 (45) |
| Subject factor from day 1 to 5 | **42** |
| BPS or > 3 or NRS > 5, | 30 (71) |
| Fatigue | 4 (10) |
| Nausea | 6 (14) |
| Patient refusal | 2 (5) |
| Medical staff factor from day 1 to 5 | **96** |
| Holiday | 60 (63) |
| Lack of staff | 10 (10) |
| No intervention | 22 (23) |
| Poor time adjustment | 4 (4) |

*F_I_O_2_* _=_ fraction of inspiratory oxygen; *SpO_2_* = saturation of percutaneous oxygen; *PEEP* = positive end expiratory pressure ventilation; *BPS* = behavioral pain scale; *NRS* = numerical rating scale.

**Supplementary Figure 1.** **Maximum level of activity from days 1 to 5**


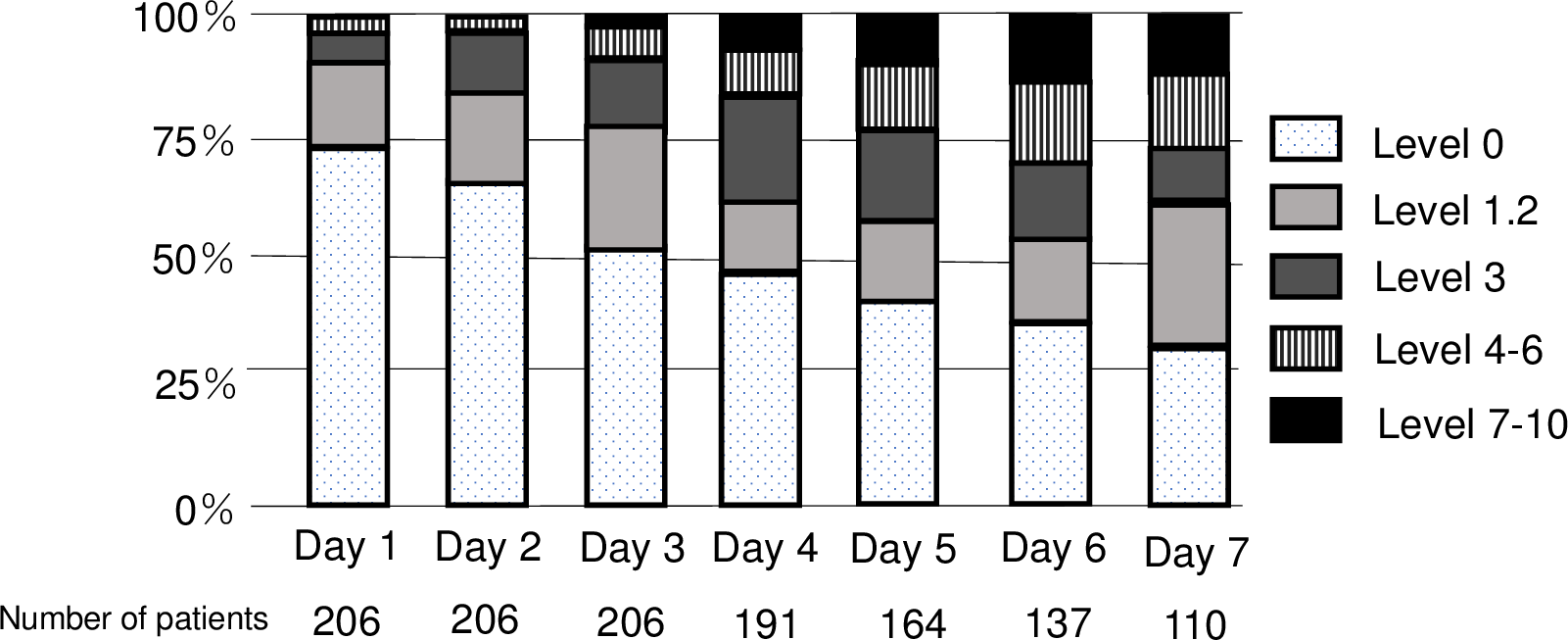


Measured using the Intensive Care Unit Mobility Scale: 0=no activity, 1=exercises in bed, 2=passively moved to the chair, 3=sitting on the edge of the bed, 4=standing, 5=transferring from bed to chair through standing, 6=marching on the spot, 7=walking with assistance of two people, 8=walking with assistance of one person, 9=walking with an assistive device, and 10=walking independently.
